# Development and validation of an artificial intelligence-based tool to detect subclinical atheroesclerosis using non-mydriatic retinal funduscopic images: the Aitheroscope project

**DOI:** 10.1093/ehjdh/ztag129

**Published:** 2026-08-10

**Authors:** Juan Torres-Macho, María Ángeles Sánchez-Uriz, Vyara Hrystova, Jesús Prada-Alonso, Jose Manuel López-Aragonés, Nuria Muñoz-Rivas, Anabel Franco-Moreno, Eva Moya-Mateo, María Pilar Ferrera-Ahijado, Eva María Duro-Perales, María Antón-Vallejo, Montserrat Saiz-González, Alberto Diez-García, Celeste Marina-Verde, Andrés Rosa-López, Inmaculada Fernández-Sotillo, Carolina Espejo-Paeres, Miguel Bravo-Prieto, Javier Corrochano del Pino, Carmen Pantoja-Zarza, María José Crespo-Carballés

**Affiliations:** Internal Medicine Department, Infanta Leonor University Hospital, Av Gran vía del este 80, Madrid 28031, Spain; Department of Medicine, Complutense University, Madrid; Occupational Health Department, Infanta Leonor University Hospital, Madrid, Spain; Ophthalmology Department, Infanta Leonor University Hospital, Madrid, Spain; Horus-ML, Alcalá Street 268, Madrid 28027, Spain; Occupational Health Department, Infanta Leonor University Hospital, Madrid, Spain; Department of Medicine, Complutense University, Madrid; Cardiovascular Risk Unit, Internal Medicine Department, Infanta Leonor University Hospital, Madrid, Spain; Department of Medicine, Complutense University, Madrid; Cardiovascular Risk Unit, Internal Medicine Department, Infanta Leonor University Hospital, Madrid, Spain; Department of Medicine, Complutense University, Madrid; Cardiovascular Risk Unit, Internal Medicine Department, Infanta Leonor University Hospital, Madrid, Spain; Occupational Health Department, Infanta Leonor University Hospital, Madrid, Spain; Occupational Health Department, Infanta Leonor University Hospital, Madrid, Spain; Occupational Health Department, Infanta Leonor University Hospital, Madrid, Spain; Occupational Health Department, Infanta Leonor University Hospital, Madrid, Spain; Occupational Health Department, Infanta Leonor University Hospital, Madrid, Spain; Ophthalmology Department, Infanta Leonor University Hospital, Madrid, Spain; Ophthalmology Department, Infanta Leonor University Hospital, Madrid, Spain; Ophthalmology Department, Infanta Leonor University Hospital, Madrid, Spain; Horus-ML, Alcalá Street 268, Madrid 28027, Spain; Horus-ML, Alcalá Street 268, Madrid 28027, Spain; Horus-ML, Alcalá Street 268, Madrid 28027, Spain; Hospital Manager, Infanta Leonor University Hospital, Madrid, Spain; Department of Medicine, Complutense University, Madrid; Ophthalmology Department, Infanta Leonor University Hospital, Madrid, Spain

**Keywords:** Artificial intelligence, Subclinical atherosclerosis, Retinal fundus imaging, Non-mydriatic retinography, Cardiovascular risk, Screening, Machine learning

## Abstract

**Aims:**

Current cardiovascular risk scores may underestimate the risk of future cardiovascular events, particularly in younger individuals with prolonged exposure to risk factors. In contrast, imaging-based detection of subclinical atherosclerosis provides a more accurate assessment of cardiovascular risk by identifying established vascular disease. We aimed to develop and prospectively validate an artificial intelligence–based tool using retinal fundus images to detect ultrasound-confirmed subclinical atherosclerosis.

**Methods and results:**

In this prospective observational study, 931 participants (mean age 52.6 years; 70.2% women) without prior cardiovascular disease underwent standardized clinical evaluation, non-mydriatic retinal imaging, and carotid and femoral ultrasound to detect subclinical atherosclerosis. A multimodal AI model integrating deep learning from retinal images with radiomic and clinical data was developed in a derivation cohort (*n* = 781) and evaluated in a held-out prospective test set (*n* = 150).

Subclinical atherosclerosis was present in 50.8% of participants. In the prospective test set, the AI model demonstrated good discrimination. In image-only mode, the model achieved an area under the curve (AUC) of 0.80 (95% CI 0.73–0.87), with sensitivity of 88.2%. In the enhanced mode incorporating clinical variables, performance improved to an AUC of 0.86 (95% CI 0.80–0.92), with sensitivity of 93.4% and a negative predictive value of 90.6%. Discrimination was higher in younger individuals and those at low-to-intermediate cardiovascular risk.

**Conclusion:**

AI-based retinal image analysis enables non-invasive detection of systemic subclinical atherosclerosis. This scalable approach may enhance early identification of high cardiovascular-risk patients, particularly in populations in whom risk is underestimated.

## Graphical Abstract

**Graphical Abstract:**
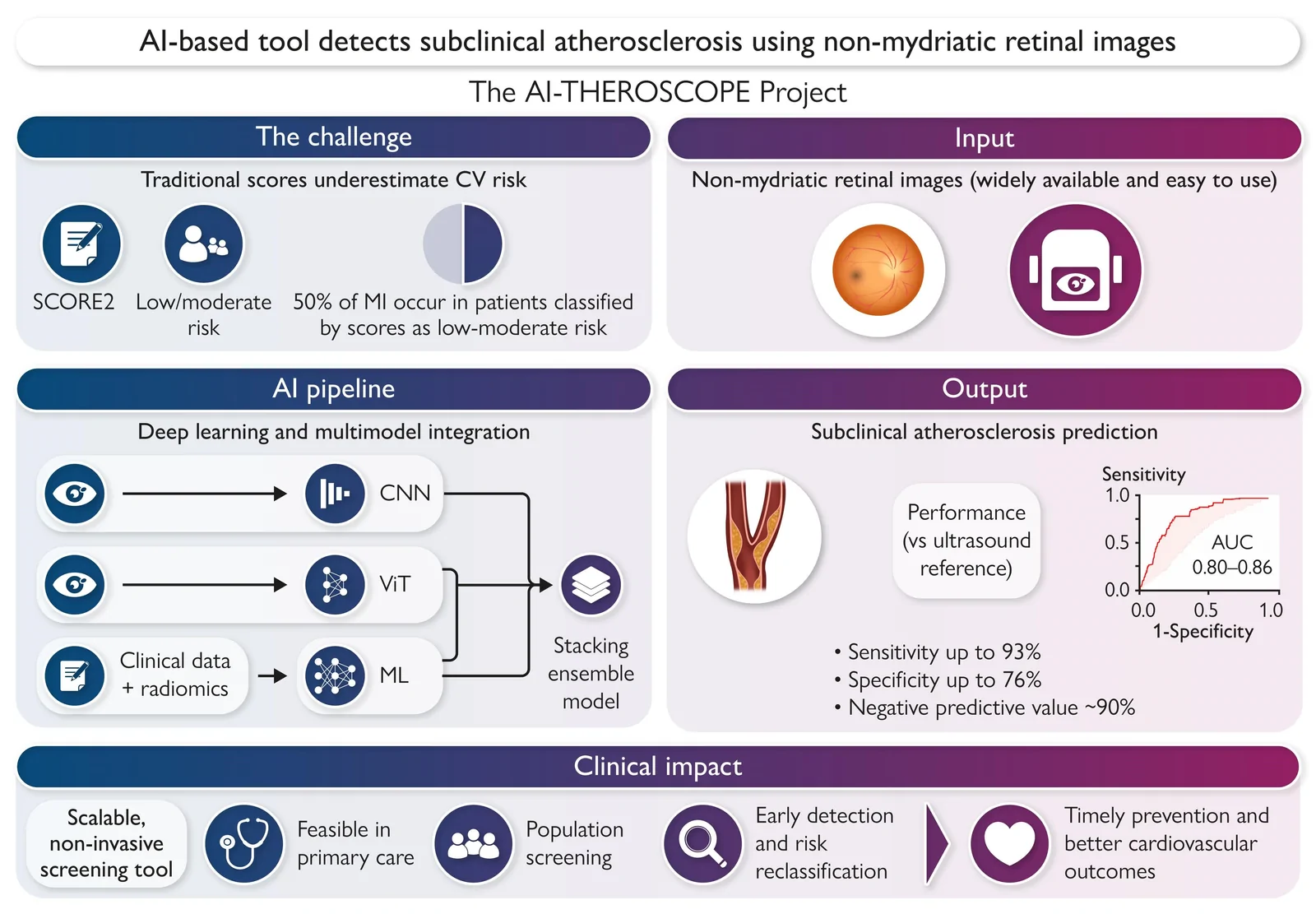

## Introduction

Cardiovascular disease remains the leading cause of morbidity and mortality worldwide despite substantial advances in prevention and treatment. Atherosclerosis develops silently over decades before the onset of clinical manifestations such as myocardial infarction or stroke. Consequently, identifying individuals with subclinical atherosclerotic disease before the occurrence of cardiovascular events represents a major opportunity for preventive intervention. Imaging-based approaches, particularly vascular ultrasound, allow direct visualization of atherosclerotic plaques in different arteries and have emerged as valuable tools for the detection of subclinical disease. The presence of ultrasound-detected atherosclerosis is strongly associated with future cardiovascular events and provides prognostic information beyond traditional cardiovascular risk factors and risk scores.^1,2^

Although clinical cardiovascular risk scores remain the cornerstone of primary prevention strategies, they estimate the probability of future events rather than directly assessing the presence of atherosclerotic disease. In Europe, SCORE2 is widely used because of its simplicity and integration into clinical guidelines. However, its reliance on a limited number of clinical variables and the strong influence of age may lead to underestimation of risk in younger individuals with prolonged exposure to cardiovascular risk factors and may limit discrimination at the individual level. Consequently, many individuals classified as low or intermediate risk already harbour subclinical atherosclerosis and may remain unidentified by conventional risk-based screening approaches.^3^

Despite its clinical value, vascular ultrasound is not ideally suited for population-wide screening because it requires trained operators, specialized equipment, and standardized acquisition protocols. These limitations have stimulated interest in developing accessible, scalable, and cost-effective imaging strategies capable of identifying individuals with underlying atherosclerotic disease before the onset of clinical events.^4^

Retinal imaging has emerged as a promising non-invasive approach for cardiovascular assessment. The retinal circulation shares anatomical, physiological, and developmental characteristics with the systemic vasculature and represents the only vascular bed that can be directly visualized *in vivo* without invasive procedures. Retinal vascular abnormalities have been associated with hypertension, diabetes mellitus, coronary artery disease, stroke, and other manifestations of systemic vascular dysfunction.^5^ Recent advances in artificial intelligence (AI) have substantially enhanced the ability to extract prognostically relevant information from retinal fundus images.^6^ Deep learning–based retinal algorithms have been shown to improve cardiovascular risk stratification beyond established clinical risk scores, particularly among individuals at intermediate risk. These findings suggest that AI-enabled retinal imaging may capture complex vascular patterns that are not readily discernible by human observers, offering a scalable and cost-effective strategy for cardiovascular screening and risk assessment.^7^

The aim of this study is to develop and validate an AI-based tool capable of identifying retinal vascular patterns associated with ultrasound-defined subclinical atherosclerosis through the analysis of static retinal images acquired using non-mydriatic fundus cameras, a widely available and easy-to-use imaging modality.

## Methods

This prospective, single-centre, observational study was conducted at a university hospital in Madrid, Spain. Eligible participants were adults aged ≥18 years who underwent cardiovascular evaluation and risk assessment at the Internal Medicine Cardiovascular Risk Unit, as well as healthcare workers and their relatives evaluated through the Occupational Health Department between January 2024 and November 2025.

The exclusion criteria included prior cardiovascular disease (acute coronary syndrome, stroke, or peripheral arterial disease), previous carotid or femoral vascular surgery or stenting, prior retinal surgery, and ocular or retinal conditions known to significantly affect retinal vasculature. These conditions included moderate or severe diabetic retinopathy, retinal vascular occlusions, advanced hypertensive retinopathy, exudative age-related macular degeneration, and macular disease of any aetiology.

After providing written informed consent, all participants underwent a standardized clinical evaluation including demographic data collection, assessment of medical history with particular emphasis on cardiovascular risk factors, measurement of blood pressure, weight and height, comprehensive blood testing (including fasting glucose, glycated haemoglobin, renal function, total cholesterol, LDL cholesterol, HDL cholesterol, triglycerides, apolipoprotein B, and lipoprotein a). Cardiovascular risk was estimated using the SCORE2 algorithm. For participants aged ≥70 years, the SCORE2-OP algorithm was applied.^8^ Risk estimation was performed using charts calibrated for low-risk Mediterranean populations. In participants younger than 40 years of age, cardiovascular risk was assessed using the PREVENT risk score.^9^ Cardiovascular risk categories were defined based on the estimated 10-year risk of cardiovascular events: low–intermediate risk (<5%), high risk (5–10%), and very high risk (>10%).

### Vascular ultrasound examination

Carotid and femoral ultrasound examinations were performed on the same day as retinal imaging using a high-frequency linear probe (6–10 MHz). Bilateral carotid arteries (common carotid artery, bifurcation, and internal carotid artery) and femoral arteries were systematically assessed in B-mode. Atherosclerotic plaque was defined according to the Mannheim consensus criteria.^10^ Ultrasound examinations were performed by trained nurses under physician supervision and interpreted in real time at the point of acquisition. All supervising physicians had extensive experience in vascular ultrasound. In cases of diagnostic uncertainty, images were independently reviewed by an external blinded committee of two vascular ultrasound experts.

### Retinal fundus imaging

Retinal fundus images were acquired using two non-mydriatic retinal cameras (TRC-NW400 and NW500; Topcon Healthcare, Tokyo, Japan). For each participant, a single macula-centred image was captured per eye, with a field of view of 45° and 50° for the respective devices. Images produced by the TRC-NW400 had a resolution of 1956 × 1934 pixels, whereas those from the NW500 had a resolution of 2676 × 2676 pixels. To ensure data privacy, all images were fully anonymized and subsequently reviewed by an ophthalmologist to exclude cases with significant retinal pathologies.

### Sample size

The study was designed in two sequential phases. In the first phase (derivation cohort), 781 participants (651 for training and 130 for internal validation) were used to develop the deep learning model through stratified cross-validation, ensuring balanced class representation across folds (approximately 51% positive prevalence in each partition). In the second phase (prospective validation), a separate held-out set of 150 participants, enrolled consecutively and never used during model development, was reserved for an independent performance evaluation.

The sample size estimation was based on two complementary criteria. First, for the assessment of model discrimination, the minimum number of participants required in the validation set was calculated using the variance formula proposed by Hanley and McNeil to ensure adequate precision of the area under the receiver operating characteristic curve (AUC).^11^ Assuming an expected AUC of 0.85, a disease prevalence of 50%, and a two-sided significance level of 0.05, a minimum of 162 participants was estimated to achieve a 95% confidence interval width of no more than 0.12 around the point estimate of the AUC. The final prospective validation set of 150 participants approached this threshold (estimated 95% CI width: 0.124), providing statistical power exceeding 99% to reject the null hypothesis of AUC ≤ 0.70, and 88% power to reject AUC ≤ 0.75, thereby ensuring sufficient precision to demonstrate clinically meaningful discrimination above previously reported benchmarks for retinal-based atherosclerosis detection. Second, the precision of sensitivity and specificity estimates was assessed using the method described by Buderer *et al.*^12^

Assuming an expected sensitivity of 90% and specificity of 85%, a minimum of 71 patients with subclinical atherosclerosis and 99 without the condition were required to estimate these parameters with a margin of error not exceeding ±7 percentage points at a 95% confidence level.

### AI models development and prospective validation

The deep learning pipeline was developed using a stacking ensemble architecture designed to integrate complementary modelling approaches that operate on heterogeneous data representations. The ensemble comprised three parallel processing paths. The first path used a dual-head convolutional neural network (CNN) that independently processed the left and right eye retinal images through shared convolutional layers to extract hierarchical spatial features. The second path used a Vision Transformer (ViT) initialized through domain-specific self-supervised pretraining. The ViT was pretrained in-house following the masked-image-modelling strategy described for RETFound,^13^ rather than using publicly released pretrained weights. Pretraining was performed on a curated corpus of 4576 publicly available retinal fundus images compiled from three open-access datasets (IDRiD, RFMiD and RFMiD 2.0;^14–16^), acquired with multiple non-mydriatic fundus camera models, and with source cohorts predominantly of South Asian origin (see Supplementary material online, *Table S5*). The resulting encoder was subsequently fine-tuned on the study dataset, capturing global context and long-range spatial relationships through self-attention mechanisms. For both image-based paths, right eye images were horizontally flipped prior to processing to standardize laterality and ensure anatomical correspondence with the left eye images. A field-of-view normalization step was applied to homogenize images acquired from the two retinography devices (45° and 50°capture angles) by centring and cropping them to a common region of interest. Images were then resized to uniform input dimensions and normalized using statistics derived from the training set.

The third path employed hand-crafted radiomic features (texture and morphological descriptors extracted from the retinal images) and, in enhanced mode, combined these features with patient demographics (age and sex) and clinical variables (hypertension, healthy diet adherence, and smoking status). These features were processed using a gradient boosting classifier to produce probability predictions by integrating image-derived and patient-level information.

**Figure 1:**
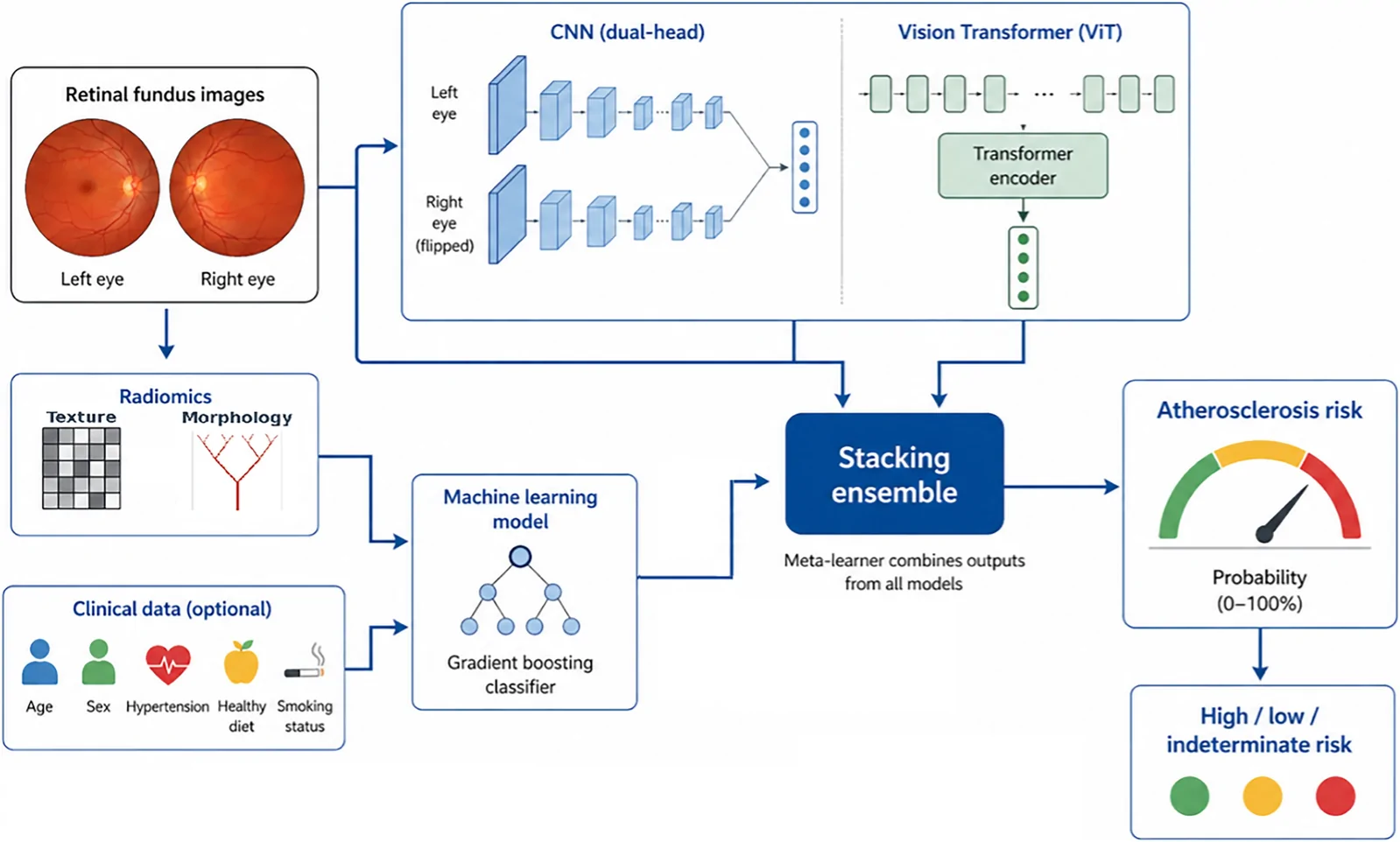
Stacking ensemble pipeline for retinal image–based atherosclerosis risk prediction. Retinal fundus images are processed through a convolutional neural network (CNN) and a Vision Transformer (ViT), while radiomic and optional clinical features are analysed using a machine learning model. Outputs from all branches are combined in a stacking ensemble to generate a final probability of atherosclerosis risk.

The outputs from all three component models were combined using a second-layer meta-model trained on held-out validation data, which learned optimal weighting across paths to produce the final calibrated probability estimate. This two-layer stacking design was selected to reduce the risk of systematic prediction errors arising from reliance on a single model family, to enable principled integration of image and tabular data within a unified framework, and to improve robustness to variations in image acquisition conditions across devices (*Figure 1*).

### Statistical analyses

Model discrimination was assessed using the area under the receiver operating characteristic curve (AUC) as the primary endpoint of the study. Secondary performance metrics included sensitivity, specificity, positive and negative predictive value, F1-score, and the area under the precision-recall curve (PRAUC). The optimal classification threshold was determined by maximizing the F1-score on the validation set and subsequently applied without modification to the held-out test set.

Ninety-five percent confidence intervals for the AUC were computed using the method of DeLong *et al*. Confidence intervals for sensitivity, specificity, and predictive values were estimated using the Clopper–Pearson exact binomial method. Confidence intervals for the F1-score and the area under the precision-recall curve were obtained by bootstrap resampling.

The system operated in two modes: basic mode using only retinal images and radiomic variables extracted from these images, and enhanced mode incorporating additional demographic and clinical parameters (age, sex, hypertension, healthy diet adherence, and smoking status).

Subgroup analyses were performed stratified by sex and age to assess potential performance disparities across clinically relevant demographic categories. Differences in AUC between subgroups were assessed using a *z*-test based on the Hanley and McNeil variance estimates. Within each subgroup, model discrimination was tested against the null hypothesis of AUC = 0.50. All statistical tests were two-sided, with a significance level of 0.05.

Gradient-weighted class activation mapping (Grad-CAM) was applied to the Vision Transformer components to generate spatial explainability visualizations identifying retinal regions that were most influential to model predictions. The contribution of each clinical variable to individual risk estimates was assessed through Shapley Additive Explanations (SHAP) analysis. Probability calibration was performed using isotonic regression fitted to the validation data to ensure that predicted probabilities accurately reflected the true likelihood of atherosclerosis across the full prediction range.

The clinical utility of the predicted probabilities was further assessed through decision curve analysis, quantifying the net benefit of each operational mode across a range of threshold probabilities relative to the default strategies of treating all or treating no patients.

The study was conducted and reported in accordance with the STROBE guidelines, given its observational design.

### Ethical considerations

This study was registered at ClinicalTrials.gov (NCT07628088) and was conducted in accordance with the Declaration of Helsinki and applicable national regulations. Patient's data were anonymized and securely stored using REDCap (Research Electronic Data Capture) database. The complete study protocol received approval from the institutional research ethics committee of Hospital Universitario Clínico San Carlos, prior to study initiation.

## Results

**Figure 2:**
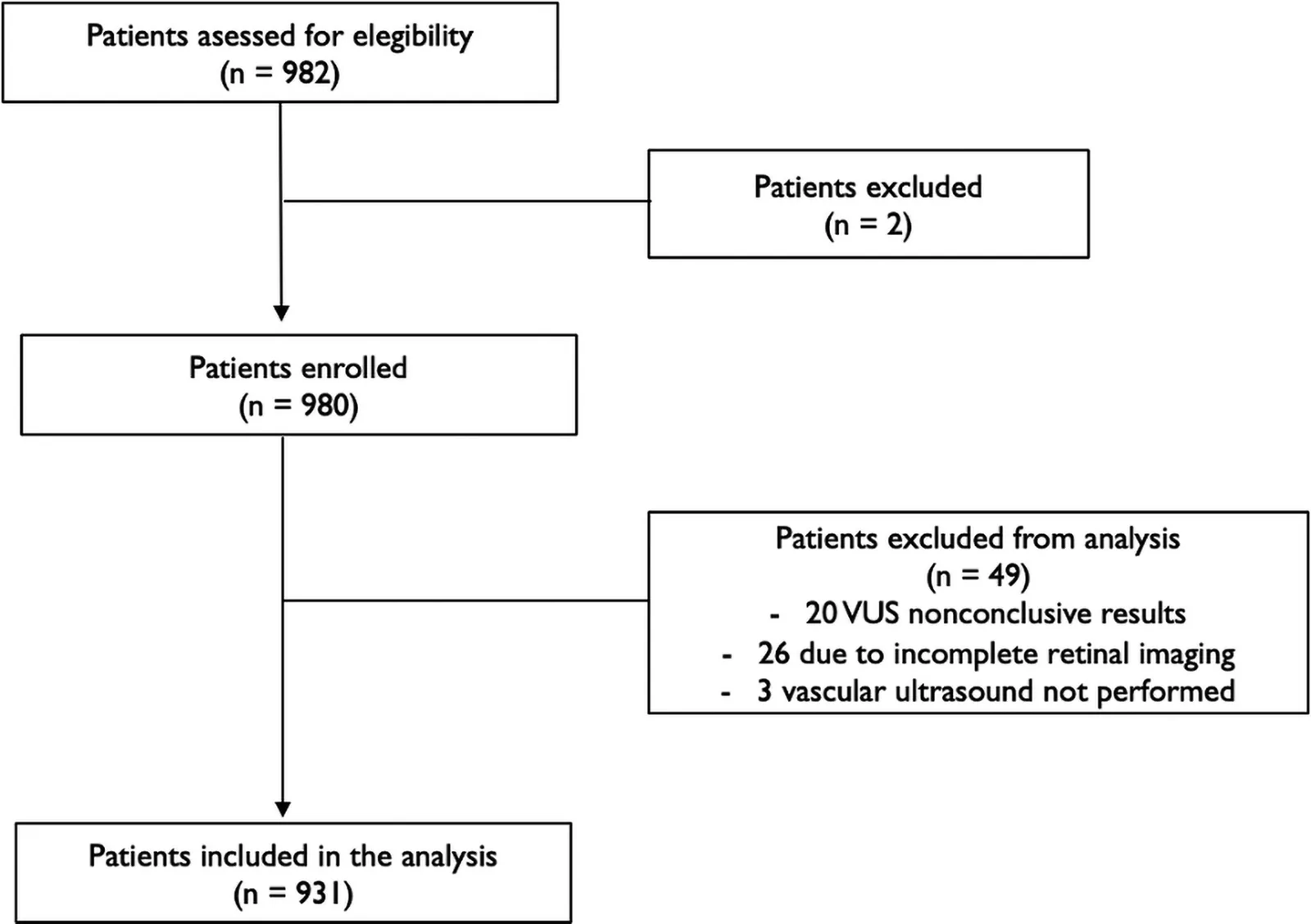
Study flowchart. VUS: vascular ultrasound.

A total of 982 patients were assessed for eligibility; two were excluded due to previous retinal disease, and 980 were finally enrolled in the study. Subsequently, three patients were excluded because vascular ultrasound was not performed, 26 due to incomplete retinal imaging in both eyes, and 20 because the presence of vascular plaque remained uncertain after expert review of the ultrasound studies. A total of 931 patients were included in the final analysis. The study flowchart is shown in *Figure 2*.

Baseline characteristics of the study population are shown in *Table 1*. Mean age was 52.6 years (range, 22–82), of whom 70.2% were women. Overall cardiovascular risk factor burden was relatively low, with hypertension present in 16.5%, dyslipidemia in 23.8%, and diabetes in 4.6% of participants. However, more than half of the population reported current or past smoking (52.5%) and nearly half were physically inactive (45.5%). Lipid levels were within the moderate range. According to the previously described cardiovascular risk stratification, 568 participants (61.0%) were classified as having low–intermediate cardiovascular risk, 143 (15.4%) as high-risk, and 171 (18.4%) as very high-risk. 30 patients (3.2%) showed significant ophthalmologic alterations (See Supplementary material online, *Table S1*).

**Table 1:** Patient’s demographics and clinical characteristics

	All patients (*n* = 931)	Neg VUS (*n* = 458)	Pos VUS (*n* = 473)	*P*
Demographic characteristics				
Age, years	52.6 (10)	48.1 (9.4)	56.9 (8.6)	<0.01
Female sex	654 (70.2)	353 (77)	301 (63.6)	< 0.01
Caucasian	846 (90.8)	419 (91.4)	427 (90.2)	0.71
Body mass index, Kg/m^2^	25.8 (4.6)	25.2 (4.4)	26.4 (4.8)	< 0.001
Systolic BP, mmHg	125.5 (15.8)	121.8 (13.9)	129 (16.6)	< 0.001
Diastolic BP, mmHg	78.5 (9.8)	77.1 (9.5)	79.9 (9.9)	< 0.001
Medical history				
Hypertension	154 (16.5)	29 (6.3)	125 (26.4)	<0.001
Dyslipidaemia	222 (23.8)	67 (14.6)	155 (32.7)	<0.001
Diabetes	43 (4.6)	9 (1.9)	34 (7.1)	<0.001
Current or past smoking	488 (52.5)	209 (45.6)	279 (58.9)	<0.001
Family history of CVD	205 (22)	102 (22.2)	103 (21.7)	0.62
Physical inactivity	424 (45.5)	199 (43.4)	225 (47.5)	0.132
Healthy diet adherence	539 (57.9)	277 (60.4)	262 (55.3)	0.183
Laboratory results				
Total cholesterol, mg/dL	196.5 (34.7)	193.7 (33.4)	199 (35.7)	0.023
HDL cholesterol, mg/dL	60.5 (14.3)	62.1 (13.6)	59.1 (14.9)	0.002
LDL cholesterol, mg/dL	116.7 (30.8)	113.8 (28.8)	119.2 (32.4)	0.009
HbA1c, %	5.5 (0.54)	5.4 (0.46)	5.6 (0.6)	< 0.001
Triglycerides, mg/dL	96.2 (55.4)	88.9 (56.5)	103.5 (53.6)	<0.001
Serum creatinine, mg/dL	0.84 (0.16)	0.83 (0.15)	0.85 (0.16)	0.008
Lipoprotein (a), mg/dL	34.4 (38.1)	33.6 (37.7)	35.3 (38.7)	0.51
Apolipoprotein B, mg/dL	92 (27)	88 (26)	94 (30)	0.2

Categorical variables are presented as number (%), and continuous variables as mean (standard deviation).

CVD, cardiovascular disease.

A total of 473 patients (50.8%) had subclinical atherosclerosis in at least one vascular territory. Plaque involvement was observed in one vascular territory in 154 participants (16.5%), two territories in 158 (17.0%), three territories in 85 (9.2%), and all four territories in 76 (8.2%). Subclinical atherosclerosis showed a relatively homogeneous distribution across vascular territories, with plaques detected in approximately 27–29% of participants in each arterial segment.

### AI tool performance

**Figure 3:**
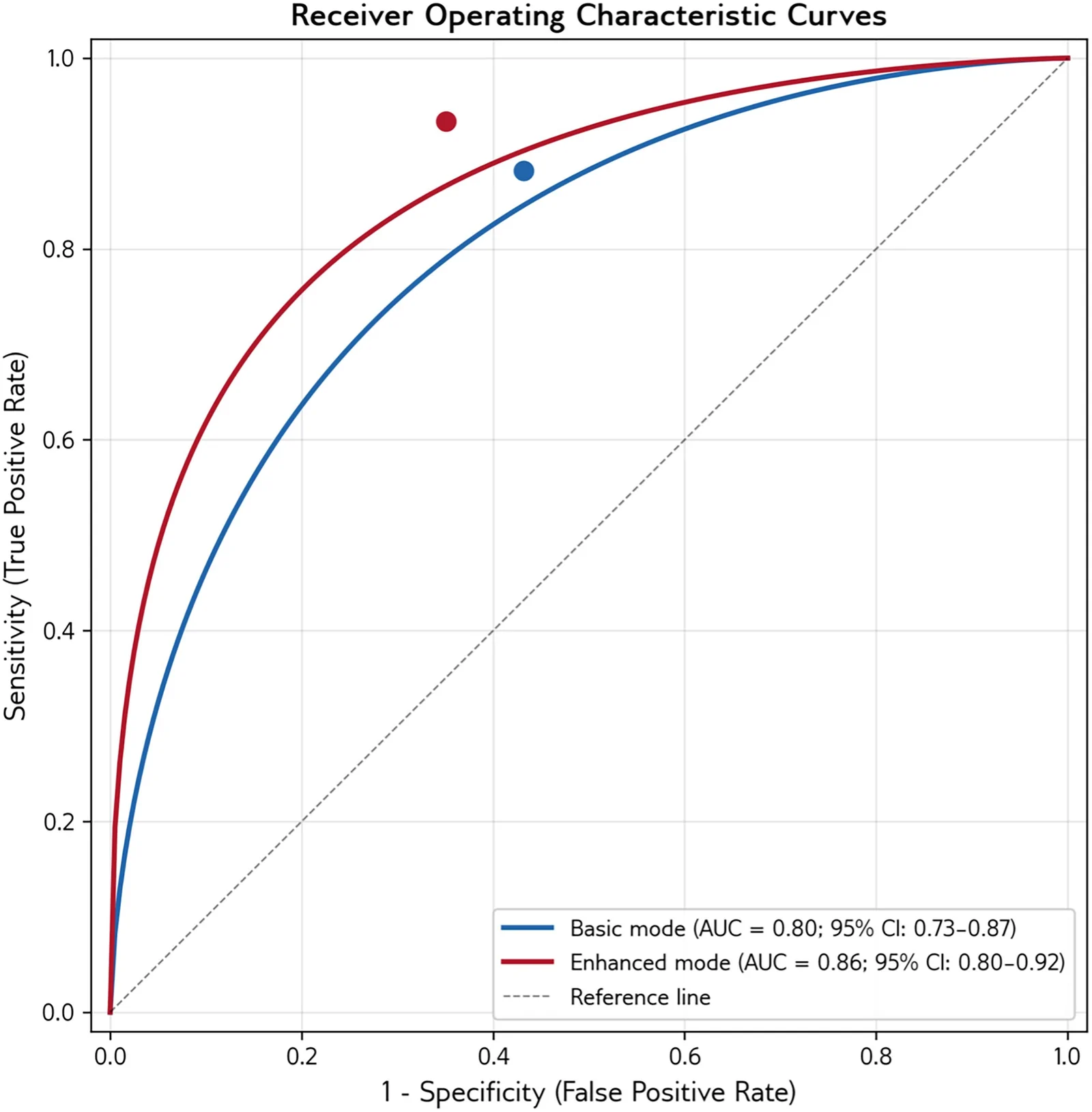
AUC of basic and enhanced modes over the held-out test set.

On the prospective held-out test set (*n* = 150; 76 positive and 74 negative for subclinical atherosclerosis), reserved exclusively for independent evaluation and fulfilling the predefined sample size requirements for sensitivity estimation, the AI tool demonstrated good discrimination for the detection of ultrasound-confirmed subclinical atherosclerosis in both operational modes. Internal cross-validation performance on the derivation cohort (*n* = 781) was consistent with test set results, supporting the stability and generalizability of the model estimates. *Figure 3* and Supplementary material online (Supplementary material online, *Table S2*) shows the receiver operating characteristic (ROC) curves for the basic and enhanced configurations.

In basic mode (only retinal images), the model achieved an AUC of 0.80 (95% CI: 0.73–0.87), with a high sensitivity of 88.2% (95% CI: 78.7–94.4%). In an enhanced mode (incorporating age, sex, hypertension, healthy diet adherence, and smoking status), discrimination improved to an AUC of 0.86 (95% CI: 0.80–0.92), with sensitivity of 93.4% (95% CI: 85.3–97.8%) and a negative predictive value of 90.6% (95% CI: 79.3–96.9%), F1-score of 0.82, precision-recall AUC of 0.85, and Brier score of 0.17.

In subgroup analyses, model performance was consistent with the overall results, with improved discrimination in the enhanced mode across most strata. According to cardiovascular risk categories, AUC increased from 0.76 to 0.82 in the low–intermediate risk group, with higher sensitivity (81.2–89.6%) and specificity (58.5–67.7%), and an NPV close to 90%. In the high-risk group, sensitivity reached 100% in both modes, although specificity was not estimable due to the high prevalence of disease (89.5%). In very high-risk individuals (100% prevalence), discrimination metrics were not applicable. In patients not assessable by cardiovascular risk scores due to incomplete clinical data required for risk-score calculation, sensitivity and NPV remained at 100% in both modes, with moderate specificity (∼57%). By age group, discrimination was highest in younger participants (≤45 years; AUC 0.85 to 0.90), with sensitivity and NPV reaching 100% in the enhanced mode, while performance was more modest in older groups (56–65 years; AUC 0.65 to 0.72), with lower specificity but higher positive predictive values, likely reflecting the high baseline prevalence of subclinical atherosclerosis in these groups. Subgroup analysis by sex showed no statistically significant difference in model discrimination between women (AUC 0.83; 95% CI: 0.74–0.92) and men (AUC 0.86; 95% CI: 0.77–0.96; *P* = 0.63 for the difference) in enhanced mode, indicating consistent performance across sexes. A subgroup analysis by fundus camera showed comparable discrimination for images acquired with the two devices (enhanced mode AUC 0.87 for the TRC-NW400 and 0.84 for the NW500), with overlapping confidence intervals and no relevant differences across the remaining metrics (*Table 2*), supporting the robustness of the model to the acquisition device. The same age-dependent pattern was observed in basic mode (*Table 2* and see Supplementary material online, *Table S3*).

**Table 2:** Complete performance metrics on the prospective test set stratified by cardiovascular risk category, age group, sex, and fundus camera

Subgroup	*n*	Prev	Basic mode							Enhanced mode						
	
			AUC	AUPRC	Sens	Spec	PPV	NPV	F1	AUC	AUPRC	Sens	Spec	PPV	NPV	F1
Overall	150	50.7%	0.80 (0.73–0.87)	0.80 (0.72–0.87)	88.2% (78.7–94.4)	56.8% (44.7–68.2)	67.7% (57.5–76.7)	82.4% (69.1–91.6)	0.77 (0.69–0.83)	0.86 (0.80–0.92)	0.85 (0.79–0.91)	93.4% (85.3–97.8)	64.9% (52.9–75.6)	73.2% (63.2–81.7)	90.6% (79.3–96.9)	0.82 (0.75–0.88)
***SCORE2 cardiovascular risk category***																
Low–intermediate	113	42.5%	0.76 (0.67–0.85)	0.70 (0.59–0.82)	81.2% (67.4–91.1)	58.5% (45.6–70.6)	59.1% (46.3–71.0)	80.9% (66.7–90.9)	0.68 (0.57–0.78)	0.82 (0.74–0.90)	0.77 (0.67–0.87)	89.6% (77.3–96.5)	67.7% (54.9–78.8)	67.2% (54.3–78.4)	89.8% (77.8–96.6)	0.77 (0.67–0.85)
High risk	19	89.5%	0.79 (0.51–1.00)	0.97 (0.88–1.00)	100.0% (80.5–100.0)	N/A	89.5% (66.9–98.7)	N/A	0.94 (0.84–0.97)	0.85 (0.62–1.00)	0.98 (0.90–1.00)	100.0% (80.5–100.0)	N/A	89.5% (66.9–98.7)	N/A	0.94 (0.84–0.97)
Very high risk	4	100%	N/A	N/A	100.0% (39.8–100.0)	N/A	100.0% (39.8–100.0)	N/A	1.00 (0.57–1.00)	N/A	N/A	100.0% (39.8–100.0)	N/A	100.0% (39.8–100.0)	N/A	1.00 (0.57–1.00)
Not assessable^a^	14	50.0%	0.72 (0.44–1.00)	0.71 (0.50–0.96)	100.0% (59.0–100.0)	57.1% (18.4–90.1)	70.0% (34.8–93.3)	100.0% (39.8–100.0)	0.82 (0.57–1.00)	0.80 (0.56–1.00)	0.80 (0.59–1.00)	100.0% (59.0–100.0)	57.1% (18.4–90.1)	70.0% (34.8–93.3)	100.0% (39.8–100.0)	0.82 (0.57–1.00)
***Age group***																
≤ 45 years	41	17.1%	0.85 (0.66–1.00)	0.59 (0.33–0.91)	85.7% (42.1–99.6)	73.5% (55.6–87.1)	40.0% (16.3–67.7)	96.2% (80.4–99.9)	0.55 (0.25–0.76)	0.90 (0.74–1.00)	0.71 (0.44–0.96)	100.0% (59.0–100.0)	79.4% (62.1–91.3)	50.0% (23.0–77.0)	100.0% (87.2–100.0)	0.67 (0.38–0.87)
46–55 years	52	48.1%	0.74 (0.60–0.88)	0.72 (0.58–0.86)	88.0% (68.8–97.5)	48.1% (28.7–68.1)	61.1% (43.5–76.9)	81.2% (54.4–96.0)	0.72 (0.58–0.84)	0.81 (0.69–0.93)	0.80 (0.67–0.92)	92.0% (74.0–99.0)	51.9% (31.9–71.3)	63.9% (46.2–79.2)	87.5% (61.7–98.4)	0.75 (0.62–0.86)
56–65 years	34	73.5%	0.65 (0.45–0.85)	0.83 (0.72–0.95)	88.0% (68.8–97.5)	33.3% (7.5–70.1)	78.6% (59.0–91.7)	50.0% (11.8–88.2)	0.83 (0.71–0.93)	0.72 (0.54–0.90)	0.87 (0.75–0.96)	92.0% (74.0–99.0)	55.6% (21.2–86.3)	85.2% (66.3–95.8)	71.4% (29.0–96.3)	0.88 (0.78–0.96)
> 65 years	23	82.6%	0.63 (0.34–0.92)	0.88 (0.77–0.98)	89.5% (66.9–98.7)	25.0% (0.6–80.6)	85.0% (62.1–96.8)	33.3% (0.8–90.6)	0.87 (0.74–0.98)	0.70 (0.44–0.96)	0.91 (0.80–0.99)	94.7% (74.0–99.9)	50.0% (6.8–93.2)	90.0% (68.3–98.8)	66.7% (9.4–99.2)	0.92 (0.81–1.00)
***Sex***																
Women	94	41.5%	0.77 (0.67–0.87)	0.70 (0.59–0.83)	87.2% (72.6–95.7)	58.2% (44.1–71.3)	59.6% (45.8–72.4)	86.5% (71.2–95.5)	0.71 (0.59–0.80)	0.83 (0.74–0.92)	0.78 (0.67–0.89)	92.3% (79.1–98.4)	69.1% (55.2–80.9)	67.9% (53.7–80.1)	92.7% (80.1–98.5)	0.78 (0.68–0.86)
Men	56	66.1%	0.79 (0.67–0.91)	0.87 (0.78–0.95)	89.2% (74.6–97.0)	52.6% (28.9–75.6)	78.6% (63.2–89.7)	71.4% (41.9–91.6)	0.84 (0.73–0.91)	0.86 (0.76–0.96)	0.92 (0.84–0.98)	94.6% (81.8–99.3)	52.6% (28.9–75.6)	79.5% (64.7–90.2)	83.3% (51.6–97.9)	0.86 (0.78–0.94)
**Fundus camera**																
TRC-NW400	77	50.7%	0.81 (0.71–0.91)	0.81 (0.70–0.91)	89.5% (75.8–97.1)	57.9% (40.8–73.7)	69.0% (54.1–80.9)	84.0% (65.1–95.6)	0.79 (0.67–0.86)	0.87 (0.79–0.95)	0.87 (0.78–0.95)	94.7% (82.7–99.4)	67.6% (51.3–82.5)	75.5% (61.1–86.7)	92.3% (76.5–99.1)	0.84 (0.75–0.91)
NW500	73	50.7%	0.79 (0.69–0.89)	0.79 (0.67–0.90)	86.8% (71.2–95.5)	55.6% (38.1–72.1)	66.2% (51.6–79.6)	80.6% (59.3–93.2)	0.75 (0.64–0.85)	0.84 (0.75–0.93)	0.84 (0.75–0.93)	91.9% (78.1–98.3)	61.1% (43.5–76.9)	71.4% (55.9–83.0)	88.5% (68.8–97.5)	0.80 (0.70–0.88)

Basic mode: retinal images only. Enhanced mode: retinal images with demographic and clinical variables. N/A: not calculable due to absence of negative cases in the subgroup.

^a^Cardiovascular risk score not assessable due to incomplete clinical data required for its calculation. 95% confidence intervals were computed using the DeLong method for the AUC, the Clopper-Pearson exact method for sensitivity, specificity and predictive values, and bootstrap resampling for the F1-score and AUPRC. Confidence intervals reaching 100% (or 0%) in small subgroups reflect the instability of estimates based on few observations rather than perfect (or absent) sensitivity/specificity.

Abbreviation: AUC, area under the ROC curve; AUPRC, area under the precision-recall curve; NPV, negative predictive value; PPV, positive predictive value; Prev, disease prevalence; Sens, sensitivity; Spec, specificity.

Analysis of false-negative cases revealed that misclassifications were concentrated among patients with limited atherosclerotic burden. In the enhanced mode, all five false negatives had plaque involvement restricted to one (*n* = 4; 80%) or two (*n* = 1; 20%) vascular territories, with no false negatives observed among patients with three or four territory disease. A similar pattern was observed in the basic mode, where all nine false negatives involved one (*n* = 7; 78%) or two (*n* = 2; 22%) territories. No patient with multi-territorial atherosclerosis (three or four vascular territories) was misclassified in either mode, suggesting that the model reliably identified individuals with the highest systemic atherosclerotic burden (see Supplementary material Supplementary material online, *Table S4*).

The mean analysis time of the AI tool was 0.13 s per patient, measured from data reception to delivery of the complete output, including probability score, categorical classification, and explainability visualizations.

**Figure 4:**
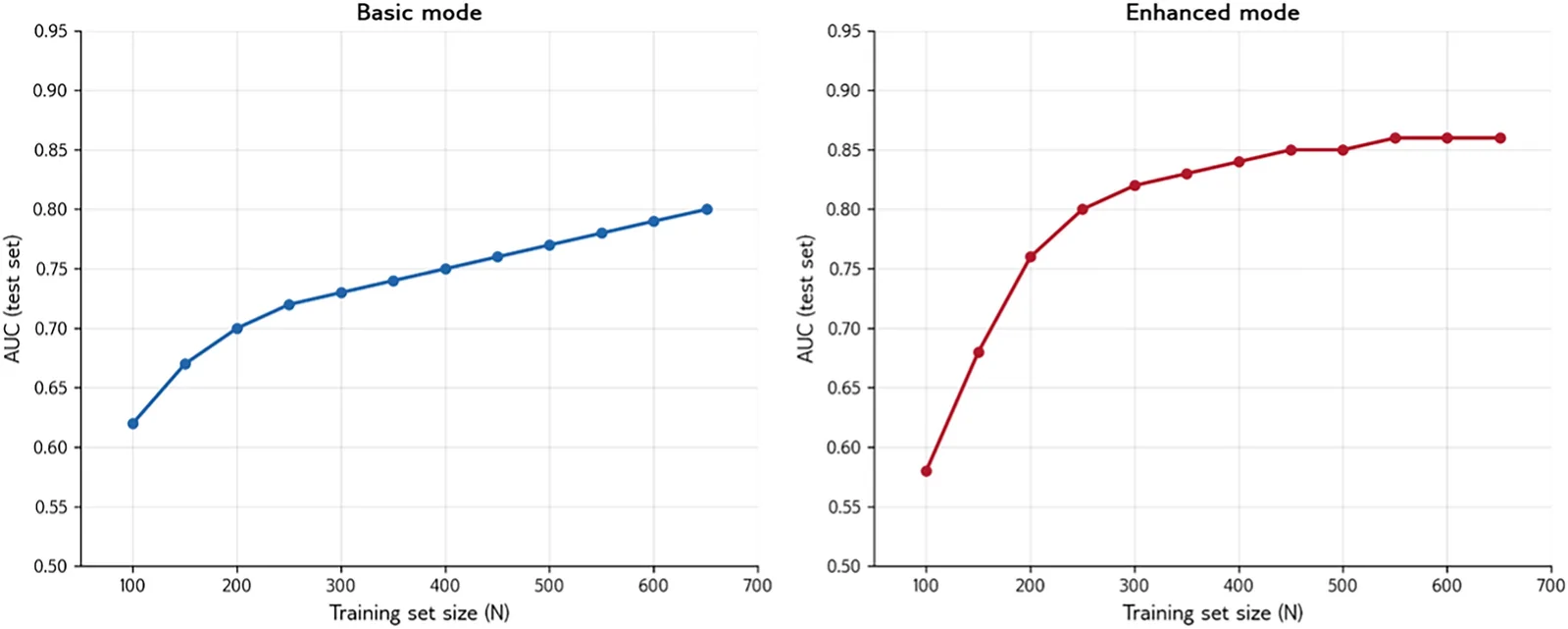
Training data saturation curves showing AUC on the held-out test set as a function of training set size for basic mode (left) and enhanced mode (right).

To evaluate whether model performance had reached a plateau with the available training data, a saturation analysis was conducted by training both model configurations on progressively larger random subsets of the training cohort (from *n* = 100 to *n* = 651) while evaluating on the fixed held-out test set (*n* = 150). As shown in *Figure 4*, the enhanced mode exhibited a steep learning curve that approached a clear plateau beyond approximately 400 training samples, suggesting that additional training data would yield diminishing returns. In contrast, the basic mode showed a more gradual and sustained upward trend, indicating that image-only performance may continue to benefit from larger training cohorts.

**Figure 5:**
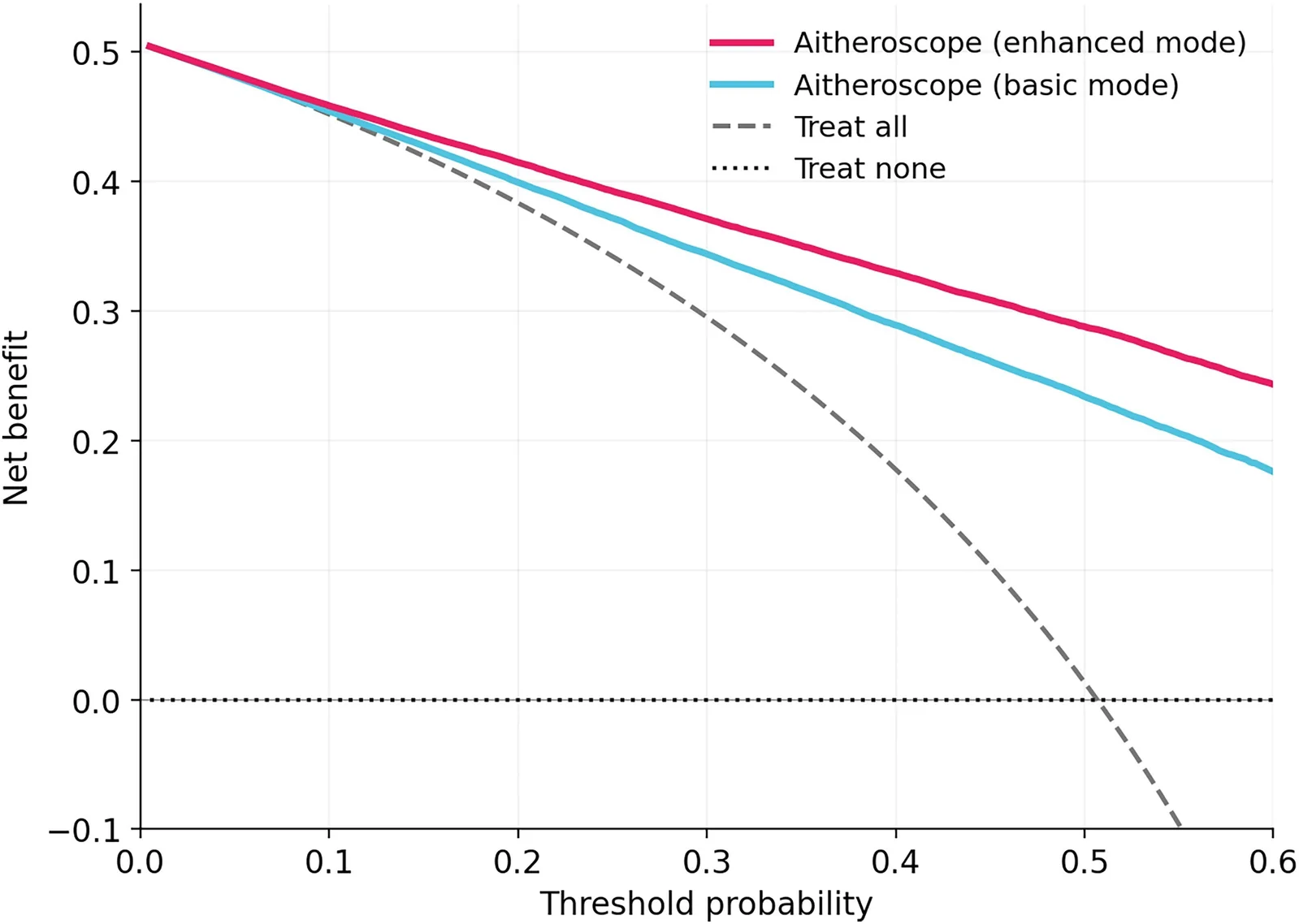
Decision curve analysis showing the net benefit of the basic and enhanced modes across a range of threshold probabilities on the held-out test set, compared with the treat-all and treat-none reference strategies.

To quantify the clinical utility of the model beyond discrimination, a decision curve analysis was performed across a range of threshold probabilities (*Figure 5*). Throughout the range of threshold probabilities relevant to a screening setting, both operational modes yielded a higher net benefit than the default strategies of treating all or treating no patients.

### Model interpretability

To support clinical interpretability, Aitheroscope incorporates two complementary explainability mechanisms. Gradient-weighted class activation mapping (Grad-CAM) was applied to the Vision Transformer components to produce spatial heatmaps identifying retinal regions most influential to the model's probability estimate. The resulting activation maps were upsampled to match the original image resolution, normalized, and rendered using a perceptually appropriate colour scale in which warm tones indicated regions of highest influence. These overlays were alpha-blended with the original retinal fundus images to allow a visual correlation between the model's attention pattern and anatomical structures.

**Figure 6:**
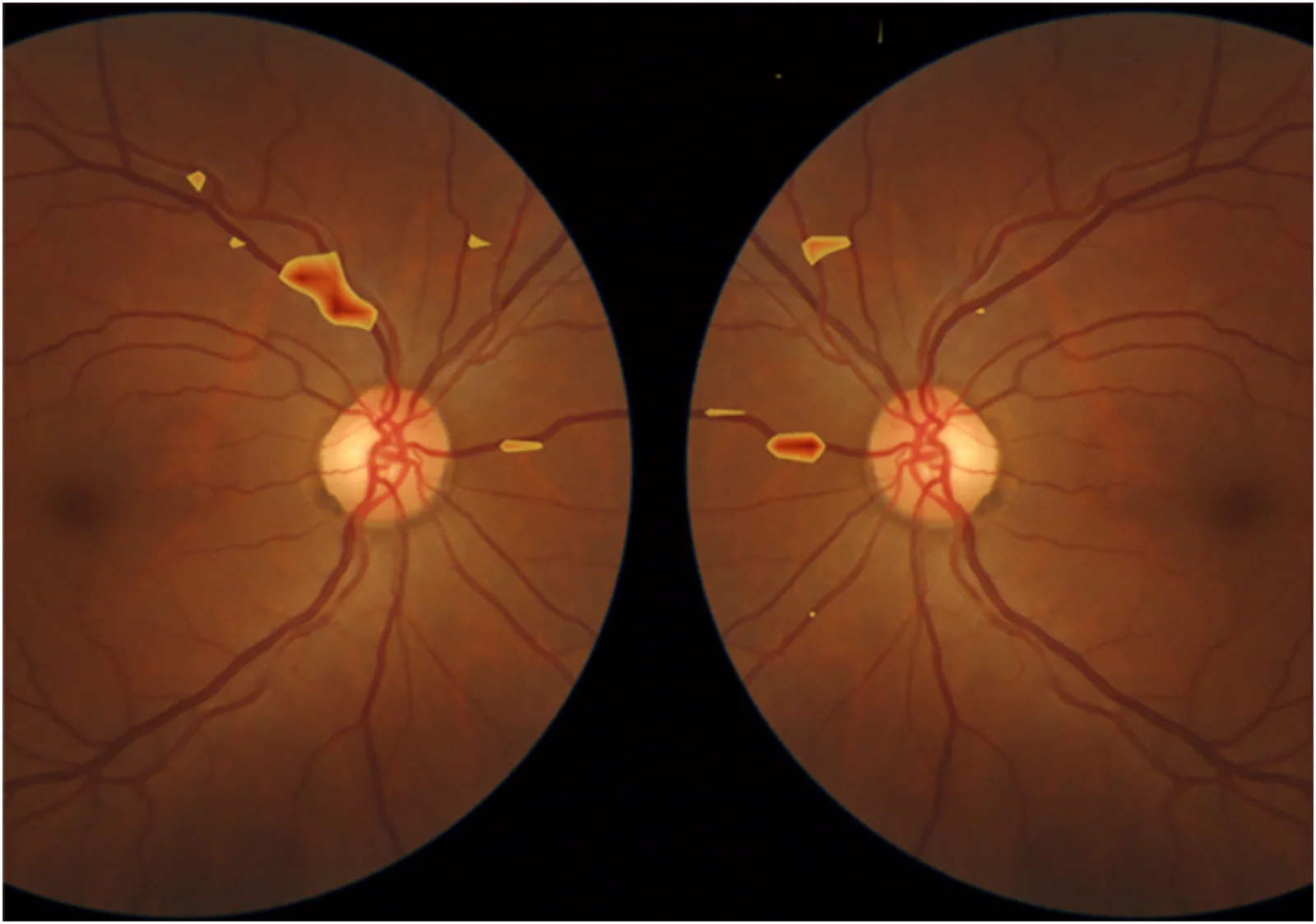
Grad-CAM explainability overlay on bilateral retinal fundus images of a representative high-risk patient. Warm colours indicate regions of highest model attention.

As shown in *Figure 6*, the model's attention was consistently directed towards the peripapillary and perimacular vascular arcades, areas where the retinal vessel calibre, tortuosity, and branching geometry reflect cumulative haemodynamic and metabolic exposure. Notably, activation was not concentrated on the optic disc or fovea themselves, but on the surrounding vascular network, supporting the biological plausibility of the model's learned features as markers of systemic vascular remodelling rather than artefactual correlations.

**Figure 7:**
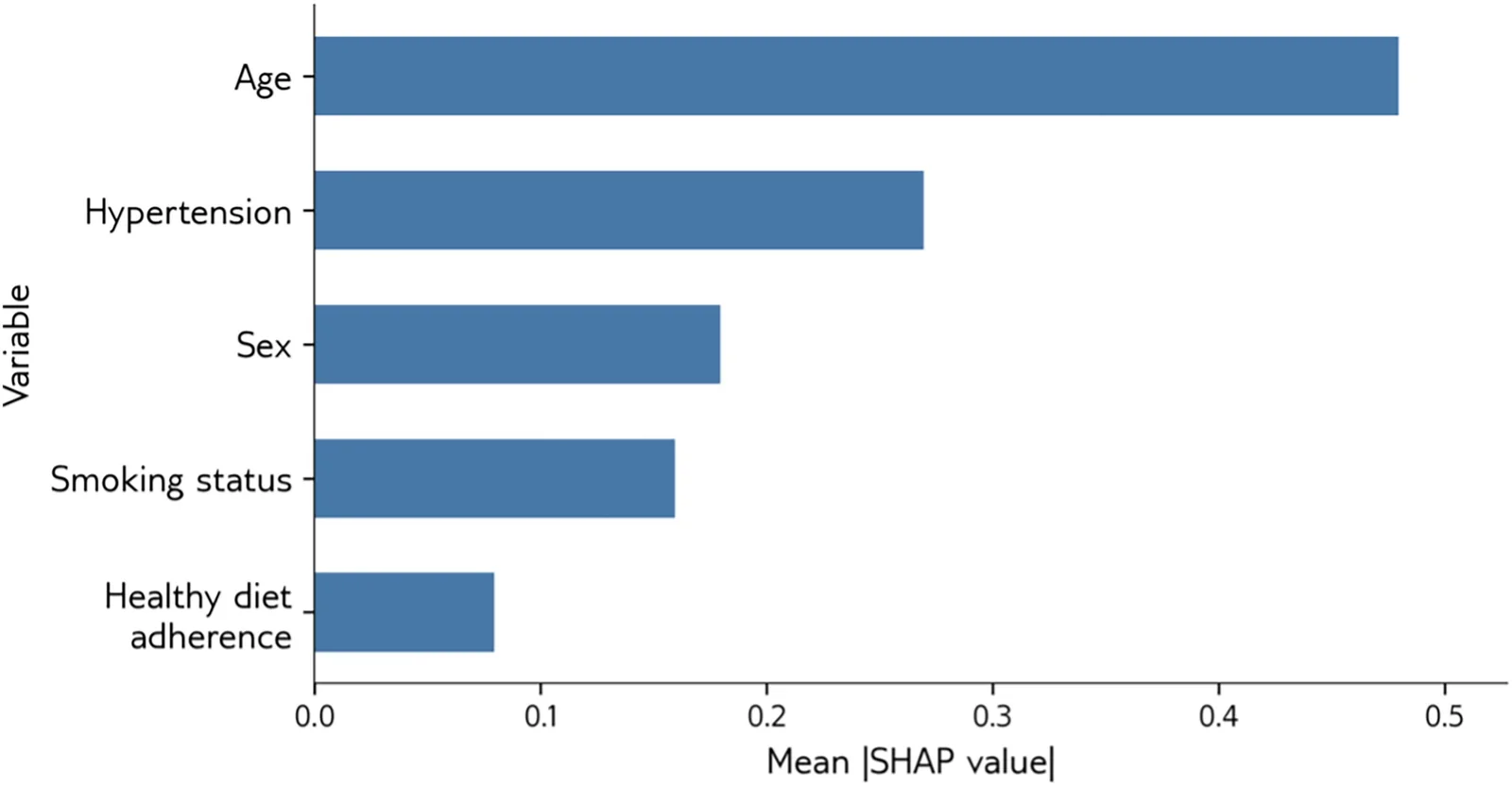
Mean absolute SHAP values for clinical variables in the enhanced mode.

In enhanced mode, SHapley Additive Explanations (SHAP) values were computed to quantify the individual contribution of each demographic and clinical variable to the patient-level risk estimate. As illustrated in *Figure 7*, age was the dominant clinical predictor, followed by hypertension, sex, smoking status, and healthy diet adherence. This ranking is consistent with the known epidemiology of subclinical atherosclerosis, in which cumulative vascular exposure, largely driven by age and hypertension, accounts for the greatest proportion of disease variance.

The combination of spatial explainability through Grad-CAM and feature-level explainability through SHAP provides healthcare professionals with a dual interpretive framework: the heatmap enables qualitative assessment of whether the algorithm focused on anatomically plausible retinal structures, while the SHAP ranking contextualizes the relative weight of modifiable and non-modifiable risk factors in the individual prediction.

## Discussion

In this prospective study, we demonstrated that deep learning analysis of retinal fundus images can identify ultrasound-confirmed subclinical atherosclerosis involving both carotid and femoral arterial territories, based on the concept of the retina as a non-invasive surrogate marker of systemic atherosclerotic disease.

Several aspects distinguish this study from prior studies. Previous deep learning approaches applied to retinal imaging have primarily focused on predicting cardiovascular risk factors or estimating global cardiovascular risk. Although these models may improve statistical discrimination, risk estimation alone does not necessarily translate into changes in clinical management at the individual level. In contrast, the detection of subclinical atherosclerosis reflects established arterial disease rather than a probabilistic risk. Contemporary cardiovascular prevention guidelines recognize imaging-detected plaques as evidence of target-organ damage and a marker of increased risk. Therefore, the identification of carotid or femoral plaque may therefore reclassify individuals into a higher risk category and support intensification of preventive strategies, including lower LDL-cholesterol targets, earlier initiation of lipid-lowering therapy, optimization of blood pressure control, and more intensive lifestyle interventions.^17–19^

Several important distinctions emerge when compared with prior similar studies. Both the original DL-FAS model developed by Chang *et al.* and the more recent foundation model-based framework proposed by Lee *et al*. were developed and validated within retrospective health screening cohorts. In contrast, Aitheroscope was evaluated within a prospectively designed framework with predefined imaging protocols and vascular endpoints. The prospective nature of Aitheroscope strengthens internal validity, reduces spectrum and information bias, and enhances clinical applicability in real-world prevention settings.^20,21^

From a discrimination standpoint, both prior studies reported comparable performance for carotid atherosclerosis detection, with AUROC values of 0.71 in Chang *et al*. and 0.71 in the best-performing foundation model configuration (DINOv2 with LoRA tuning) in Lee *et al*. In both cases, specificity remained modest (0.40 and 0.44, respectively), reflecting a performance profile driven largely by high sensitivity (0.89 and 0.87). Aitheroscope basic mode, which uses only retinal images as input data, demonstrated superior discrimination in prospective validation (AUROC 0.80), while preserving very high sensitivity (88%) and meaningfully improving specificity (57%). When demographic and clinical variables were incorporated (enhanced mode), discrimination improved further to an AUROC of 0.86, with sensitivity of 93% and specificity of 65%. Precision–recall performance was also substantially higher (PRAUC 0.85 vs. 0.57 in DL-FAS), resulting in a more favourable positive predictive value and F1-score. This reflects a more balanced and clinically actionable trade-off between detection and overdiagnosis. Importantly, the very high negative predictive value positions Aitheroscope as a robust rule-out screening tool, supporting scalable population-level or selective vascular screening strategies.

A further consideration concerns the broader shift in medical AI towards large, general-purpose foundation models, recently articulated in the call for globally representative retinal foundation models.^13^ Our approach is conceptually positioned differently. Rather than pursuing a single general-purpose model, Aitheroscope was designed as a task-specific tool optimized for one clinically defined endpoint: the detection of subclinical atherosclerosis validated against vascular ultrasound. Within this framework, the principles underlying foundation models are most useful as a means of initialization rather than as an end in themselves. In our case, this initialization was obtained by replicating a self-supervised, domain-specific pretraining strategy on a curated set of retinal images, rather than by adapting an existing pretrained foundation model; both routes are equally valid and serve the same purpose, namely reducing the amount of labelled, task-specific data required for a specialized model to reach clinically meaningful performance. Consistent with this, a comparatively modest pretraining set was sufficient to support strong prospective discrimination once the specialized model was trained on the target task.

In addition to discrimination metrics, differences in vascular reference standards are clinically relevant. Both Chang *et al*. and Lee *et al*. defined atherosclerosis using carotid intima–media thickness and/or carotid plaque as the sole reference standard. Although carotid assessment is well established, it may not fully capture the systemic atherosclerotic burden. The detection of femoral plaques has been shown to provide incremental prognostic information beyond carotid assessment alone and is strongly associated with future cardiovascular events.^22,23^ By targeting polyvascular plaque, Aitheroscope aligns more closely with a clinically meaningful phenotype of established systemic disease rather than isolated carotid involvement.

Aitheroscope demonstrated its greatest clinical utility in low-to-intermediate risk individuals and younger populations, where traditional risk scores are less accurate. Its consistently high sensitivity and negative predictive value support its role as a screening tool to exclude subclinical atherosclerosis, whereas its limited specificity suggests that confirmatory imaging remains necessary in positive cases.

The age-stratified results warrant careful interpretation. Discrimination measured by AUC was highest in the youngest participants (≤45 years), a stratum with low disease prevalence (17.1%). Because the area under the precision-recall curve is sensitive to the size of the positive class, AUPRC is reported alongside AUC for every subgroup (*Table 2*). In the ≤45-year group the AUPRC was lower than the AUC, reflecting the difficulty of sustaining high precision when positives are scarce; this is consistent with the lower positive predictive value observed in this stratum and is an expected property of the metric rather than evidence of poorer discrimination, as specificity and negative predictive value remained among the highest in the cohort. Conversely, in higher-prevalence strata the AUPRC was high. Reporting both metrics across all strata, including those where results were less favourable, provides a balanced picture: in low-prevalence settings the model retains strong rule-out capacity (high sensitivity and negative predictive value), which is the property most relevant to its intended screening use, while the more modest precision is consistent with the low pre-test probability of disease in these individuals.

The retinal vasculature provides a unique, non-invasive window into systemic cardiovascular health because it shares key anatomical and physiological features with the coronary and cerebral circulations. Retinal microvessels reflect the cumulative effects of haemodynamic stress, metabolic disturbances, and systemic inflammation, serving as a surrogate marker of endothelial dysfunction. Their dependence on local autoregulatory mechanisms makes the retina particularly sensitive to vascular injury, explaining the well-established association between retinal abnormalities and stroke, carotid atherosclerosis, ischaemic heart disease, and cardiovascular mortality.^24,25^

From a pathophysiological perspective, the retina shares with larger arterial territories key haemodynamic mechanisms involved in atherogenesis. At vascular bifurcations, regions of low and oscillatory shear stress promote endothelial dysfunction, facilitating lipid accumulation and vascular remodelling. In the retinal microcirculation, these processes manifest as changes in vessel calibre, tortuosity, and capillary rarefaction, whereas in the macrovascular compartment they lead to the formation and progression of atherosclerotic plaques. These observations support the concept that retinal vascular changes mirror shared biological pathways including endothelial dysfunction, chronic inflammation, and vascular remodelling that operate across vascular beds and may precede overt macrovascular disease, reinforcing the role of the retina as a non-invasive marker of systemic vascular damage.^26–30^ Deep learning algorithms can identify complex spatial patterns that are not detectable on conventional ophthalmological examination but are biologically linked to macrovascular plaque formation.^31–33^

The detection of subclinical atherosclerosis provides incremental clinical value beyond conventional risk estimation by identifying individuals with established arterial disease who can be directly reclassified into at least a high-risk category, with immediate implications for management and timely intensification of preventive strategies. In this context, our findings support the feasibility of a simple, scalable, and non-invasive care pathway based on retinal imaging. Non-mydriatic fundus cameras are widely available in primary care and have proven reliable for diabetic retinopathy screening, requiring minimal operator expertise and enabling rapid image acquisition.^34,35^ This AI tool could serve as a first-line strategy to identify individuals who warrant targeted vascular imaging and could facilitate large-scale screening or, more realistically, targeted case-finding among young individuals classified as low or intermediate risk by traditional scores, as well as in populations with an increased cardiovascular risk due to underlying conditions, such as people living with HIV, patients with systemic inflammatory diseases, or those with a history of cancer.^36–38^

### Study limitations

Our findings should be interpreted in light of several limitations. Although prospective, this was a single-centre study conducted in a selected population of healthcare workers and their relatives, which may limit the generalizability of the findings. This recruitment strategy may also have introduced selection bias, as healthcare workers can differ from the general population in terms of health awareness, healthcare access, lifestyle factors, and cardiovascular risk profiles. However, it also provided important methodological advantages by enabling a standardized screening protocol and the systematic acquisition of high-quality paired retinal imaging and vascular ultrasound data using existing hospital resources.

Another major limitation of this study is the absence of external validation. Although the model demonstrated consistent performance in the internal validation cohort, it was developed and validated in a relatively homogeneous population recruited from a single institution, with retinal images acquired using standardized protocols and equipment. Consequently, its performance and generalizability may differ across populations with different ethnic backgrounds, cardiovascular risk profiles, disease prevalence, healthcare settings, imaging platforms, and image acquisition protocols. Furthermore, the present study evaluated the detection of ultrasound-defined subclinical atherosclerosis rather than future cardiovascular events. Therefore, prospective longitudinal studies and external validation in geographically and clinically diverse cohorts are required to confirm the robustness, reproducibility, transportability, and clinical utility of the proposed approach before widespread implementation.

Although coronary calcium score (CAC) is an established marker of coronary atherosclerotic burden and has demonstrated clinical utility in cardiovascular risk stratification, vascular ultrasound was selected as the reference standard because it is radiation-free, widely available, and capable of detecting both carotid and femoral plaque, including earlier manifestations of atherosclerotic disease that may precede coronary calcification.^39^

In conclusion, Aitheroscope demonstrates that deep learning applied to retinal fundus imaging can prospectively identify systemic, multiterritorial subclinical atherosclerosis. By shifting the paradigm from probabilistic risk estimation to direct disease detection, this approach may enable clinically actionable risk reclassification and support more precise and earlier cardiovascular prevention strategies in the future. Importantly, it also outlines a plausible and partially implemented care pathway, leveraging the existing infrastructure of non-mydriatic fundus cameras already deployed in primary care for diabetic retinopathy screening, thereby facilitating their scalable integration into routine clinical practice.

## Supplementary Material

ztag129_Supplementary_Data

## Data Availability

Data cannot be shared publicly because the algorithm described in this study is subject to a pending patent application and is under development as a medical device.

## Lead author biography

**Juan Torres Macho** is an Internal Medicine specialist based in Madrid with extensive experience in clinical care, teaching, and research. He works in a hospital setting, managing complex patients and contributing to the training of medical students and residents. His research has focused on clinical ultrasound, COVID-19, and thromboembolic disease. In recent years, he has expanded his work to include radiomics and artificial intelligence applied to medical imaging and clinical decision-making.

## References

[1] Ibanez B, Fernández-Ortiz A, Fernández-Friera L, García-Lunar I, Andrés V, Fuster V. Progression of early subclinical atherosclerosis (PESA) study: JACC focus seminar 7/8. J Am Coll Cardiol 2021;78:156–179. 34238438 10.1016/j.jacc.2021.05.011

[2] Nicolaides AN, Panayiotou AG, Griffin M, Tyllis T, Bond D, Georgiou N, et al Arterial ultrasound testing to predict atherosclerotic cardiovascular events. J Am Coll Cardiol 2022;79:1969–1982. 35589158 10.1016/j.jacc.2022.03.352

[3] Graham IM, Di Angelantonio E, Visseren F, De Bacquer D, Ference BA, Timmis A, et al Systematic coronary risk evaluation (SCORE): JACC focus seminar 4/8. J Am Coll Cardiol 2021;77:3046–3057. 34140109 10.1016/j.jacc.2021.04.052

[4] Johri AM, Nambi V, Naqvi TZ, Feinstein SB, Kim ESH, Park MM, et al Recommendations for the assessment of carotid arterial plaque by ultrasound for the characterization of atherosclerosis and evaluation of cardiovascula risk: from the American society of echocardiography. J Am Soc Echocardiogr 2020;33:917–933. 32600741 10.1016/j.echo.2020.04.021

[5] van der Heide FC, Zamoum Y, Ounissi M, Berendschot TT, Cheung CY, Koronyo-Hamaoui M, et al Quantification of retinal microvascular imaging features from fundus photos in ocular and systemic disease: a framework for standardization. Prog Retin Eye Res 2026;112:101461. 41871655 10.1016/j.preteyeres.2026.101461

[6] Arnould L, Meriaudeau F, Guenancia C, Germanese C, Delcourt C, Kawasaki R, et al Using artificial intelligence to analyse the retinal vascular network: the future of cardiovascular risk assessment based on oculomics? a narrative review. Ophthalmol Ther 2023;12:657–674. 36562928 10.1007/s40123-022-00641-5 PMC10011267

[7] Wong DYL, Lam MC, Ran A, Cheung CY. Artificial intelligence in retinal imaging for cardiovascular disease prediction: current trends and future directions. Curr Opin Ophthalmol 2022;33:440–446. 35916571 10.1097/ICU.0000000000000886

[8] SCORE2 working group and ESC Cardiovascular risk collaboration . SCORE2 risk prediction algorithms: new models to estimate 10-year risk of cardiovascular disease in Europe. Eur Heart J 2021;42:2439–2454. 34120177 10.1093/eurheartj/ehab309 PMC8248998

[9] Khan SS, Matsushita K, Sang Y, Ballew SH, Grams ME, Surapaneni A, et al Development and validation of the American Heart Association's PREVENT equations. Circulation 2024;149:430–449. 37947085 10.1161/CIRCULATIONAHA.123.067626 PMC10910659

[10] Touboul PJ, Hennerici MG, Meairs S, Adams H, Amarenco P, Bornstein N, et al Mannheim carotid intima-media thickness and plaque consensus (2004-2006-2011). an update on behalf of the advisory board of the 3rd, 4th and 5th watching the risk symposia, at the 13th, 15th and 20th European Stroke Conferences, Mannheim, Germany, 2004, Brussels, Belgium, 2006, and Hamburg, Germany, 2011. Cerebrovasc Dis 2012;34:290–296. 23128470 10.1159/000343145 PMC3760791

[11] Hanley JA, McNeil BJ. The meaning and use of the area under a receiver operating characteristic (ROC) curve. Radiology 1982;143:29–36. 7063747 10.1148/radiology.143.1.7063747

[12] Buderer NMF . Statistical methodology: I. Incorporating the prevalence of disease into the sample size calculation for sensitivity and specificity. Acad Emerg Med 1996;3:895–900. 8870764 10.1111/j.1553-2712.1996.tb03538.x

[13] Zhou Y, Chia MA, Wagner SK, Ayhan MS, Williamson DJ, Struyven RR, et al A foundation model for generalizable disease detection from retinal images. Nature 2023;622:156–163. 37704728 10.1038/s41586-023-06555-x PMC10550819

[14] Porwal P, Pachade S, Kamble R, Kokare M, Deshmukh G, Sahasrabuddhe V, et al Indian diabetic retinopathy image dataset (IDRiD): a database for diabetic retinopathy screening research. Data (Basel) 2018;3:25.

[15] Pachade S, Porwal P, Thulkar D, Kokare M, Deshmukh G, Sahasrabuddhe V, et al Retinal Fundus multi-disease image dataset (RFMiD): a dataset for multi-disease detection research. Data (Basel) 2021;6:14.

[16] Panchal S, Naik A, Kokare M, Pachade S, Naigaonkar R, Phadnis P, et al Retinal Fundus multi-disease image dataset (RFMiD) 2.0: a dataset of frequently and rarely identified diseases. Data (Basel) 2023;8:29.

[17] Laclaustra M, Casasnovas JA, Fernández-Ortiz A, Fuster V, León-Latre M, Jiménez-Borreguero LJ. Femoral and carotid subclinical atherosclerosis association with risk factors and coronary calcium: The AWHS Study. J Am Coll Cardiol 2016;67:1263–1274. 26988945 10.1016/j.jacc.2015.12.056

[18] Brunner G, Virani SS, Sun W, Liu L, Dodge RC, Nambi V, et al Associations between carotid artery plaque burden, plaque characteristics, and cardiovascular events: the ARIC carotid magnetic resonance imaging study. JAMA Cardiol 2021;6:79–86. 33206125 10.1001/jamacardio.2020.5573 PMC7675218

[19] Aparicio A, Ball RE, Yiallourou J, Venkataraman S, Marwick P, Carrington T, et al Imaging-guided evaluation of subclinical atherosclerosis to enhance cardiovascular risk prediction in asymptomatic low-to-intermediate risk individuals: a systematic review. Prev Med 2021;153:106819. 34599926 10.1016/j.ypmed.2021.106819

[20] Lee H, Kim J, Kwak S, Rehman A, Park SM, Chang J. Optimizing retinal images based carotid atherosclerosis prediction with explainable foundation models. NPJ Digit Med 2025;8:582. 41028180 10.1038/s41746-025-01957-9 PMC12484881

[21] Chang J, Ko A, Park SM, Choi S, Kim K, Kim SM, et al Association of cardiovascular mortality and deep learning-funduscopic atherosclerosis score derived from retinal Fundus images. Am J Ophthalmol 2020;217:121–130. 32222370 10.1016/j.ajo.2020.03.027

[22] Davidsson L, Fagerberg B, Bergström G, Schmidt C. Ultrasound-assessed plaque occurrence in the carotid and femoral arteries are independent predictors of cardiovascular events in middle-aged men during 10 years of follow-up. Atherosclerosis 2010;209:469–473. 19897198 10.1016/j.atherosclerosis.2009.10.016

[23] López-Melgar B, Fernández-Friera L, Oliva B, García-Ruiz JM, Peñalvo JL, Gómez-Talavera S. Subclinical atherosclerosis burden by 3D ultrasound in mid-life: The PESA Study. J Am Coll Cardiol 2017;70:301–313. 28705310 10.1016/j.jacc.2017.05.033

[24] Kur J, Newman EA, Chan-Ling T. Cellular and physiological mechanisms underlying blood flow regulation in the retina and choroid in health and disease. Prog Retin Eye Res 2012;31:377–406. 22580107 10.1016/j.preteyeres.2012.04.004 PMC3418965

[25] Liew G, Wang JJ, Mitchell P, Wong TY. Retinal vascular imaging: a new tool in microvascular disease research. Circ Cardiovasc Imaging 2008;1:156–161. 19808533 10.1161/CIRCIMAGING.108.784876

[26] Wong TY, Klein R, Sharrett AR, Duncan BB, Couper DJ, Tielsch JM, et al Retinal arteriolar narrowing and risk of coronary heart disease in men and women. The Atherosclerosis Risk in Communities Study. JAMA 2002;287:1153–1159. 11879113 10.1001/jama.287.9.1153

[27] Wong TY, Klein R, Couper DJ, Cooper LS, Shahar E, Hubbard LD, et al Retinal microvascular abnormalities and incident stroke: the Atherosclerosis Risk in Communities Study. Lancet 2001;358:1134–1140. 11597667 10.1016/S0140-6736(01)06253-5

[28] Wong TY, Klein R, Nieto FJ, Klein BE, Sharrett AR, Meuer SM, et al Retinal microvascular abnormalities and 10-year cardiovascular mortality: a population-based case-control study. Ophthalmology 2003;110:933–940. 12750093 10.1016/S0161-6420(03)00084-8

[29] Chiu JJ, Chien S. Effects of disturbed flow on vascular endothelium: pathophysiological basis and clinical perspectives. Physiol Rev 2011;91:327–387. 21248169 10.1152/physrev.00047.2009 PMC3844671

[30] Gimbrone MA Jr, García-Cardeña G. Endothelial cell dysfunction and the pathobiology of atherosclerosis. Circ Res 2016;118:620–636. 26892962 10.1161/CIRCRESAHA.115.306301 PMC4762052

[31] Flammer J, Konieczka K, Bruno RM, Virdis A, Flammer AJ, Taddei S. The eye and the heart. Eur Heart J 2013;34:1270–1278. 23401492 10.1093/eurheartj/eht023 PMC3640200

[32] Klein R, Sharrett AR, Klein BE, Chambless LE, Cooper LS, Hubbard LD, et al Are retinal arteriolar abnormalities related to atherosclerosis? : The Atherosclerosis Risk in Communities Study. Arterioscler Thromb Vasc Biol 2000;20:1644–1650. 10845884 10.1161/01.atv.20.6.1644

[33] Allon R, Aronov M, Belkin M, Maor E, Shechter M, Fabian ID. Retinal microvascular signs as screening and prognostic factors for cardiac disease: a systematic review of current evidence. Am J Med 2021;134:36–47.e7. 32861624 10.1016/j.amjmed.2020.07.013

[34] American Diabetes Association Professional Practice Committee for Diabetes* . Retinopathy, neuropathy and foot care: Standards of Care in Diabetes-2026. Diabetes Care 2026;49:S261–S276. 41358886 10.2337/dc26-S012 PMC12690177

[35] Liu SL, Gonder JR, Owrangi E, Klar NS, Hramiak IM, Uvarov A, et al Effectiveness of nonmydriatic ultra-widefield retinal imaging to screen for diabetic eye disease: a randomized controlled trial (clearsight). Diabetes Care 2023;46:399–407. 36469332 10.2337/dc22-0713

[36] Zhang L, Iliescu C, Ferencik M, Finamore V, Beavers C, Patel AR, et al Coronary atherosclerosis in patients with cancer and survivors: a scientific statement from the American Heart Association. Circulation 2026;153:e916–e933. 41657216 10.1161/CIR.0000000000001391

[37] Kwok M, Dandapani H, Ibrahim N, Bloomfield GS, Patel S, Ahmed A, et al Estimating the degree of cardiovascular disease risk under-prediction in people living with HIV by existing risk models: an updated meta-analysis. AIDS 2026;40:1097–1102. 41873978 10.1097/QAD.0000000000004492

[38] Conrad N, Verbeke G, Molenberghs G, Goetschalckx L, Callender T, Cambridge G, et al Autoimmune diseases and cardiovascular risk: a population-based study on 19 autoimmune diseases and 12 cardiovascular diseases in 22 million individuals in the UK. Lancet 2022;400:733–743. 36041475 10.1016/S0140-6736(22)01349-6

[39] Son J, Shin JY, Chun EJ, Jung KH, Park KH, Park SJ. Predicting high coronary artery calcium score from retinal Fundus images with deep learning algorithms. Transl Vis Sci Technol 2020;9:28. 10.1167/tvst.9.2.28 PMC7410115 33184590

