# Supplementary material for: Development and validation of an artificial intelligence-based tool to detect subclinical atheroesclerosis using non-mydriatic retinal funduscopic images: the Aitheroscope project
Source: Eur Heart J Digit Health. 2026 Aug 10;7(7):ztag129. doi: 10.1093/ehjdh/ztag129 (PMC13492337; doi:10.1093/ehjdh/ztag129)
Supplement: Supplementary file 1 [file ztag129_supplementary_data.docx]

**Table S1. Classification of retinal findings.** Summary of major retinal conditions identified in the dataset

| **Ophthalmologic alterations** | **N** |
| --- | --- |
| Macular disease | 13 |
| Optic nerve disease | 7 |
| Cataract | 6 |
| Congenital malformations | 2 |
| Nevus | 2 |

**Table S2.** Area under the ROC curve (95% CI) across data partitions.

| **Moda** | **Training set (n = 651)** | **Validation set (n = 130)** | **Test set (n = 150)** |
| --- | --- | --- | --- |
| Basic | 0.86 (0.83–0.89) | 0.81 (0.74–0.88) | 0.80 (0.73–0.87) |
| Enhanced | 0.85 (0.82–0.88) | 0.84 (0.77–0.91) | 0.86 (0.80–0.92) |

**Table S3. Model sensitivity stratified by the number of affected vascular territories**

To assess whether false-negative classifications were associated with the extent of atherosclerotic disease, model sensitivity was evaluated according to the number of vascular territories with confirmed plaque on ultrasound. In both operational modes, misclassifications were concentrated among patients with limited atherosclerotic burden. No false negatives were observed among patients with disease involving three or four vascular territories in either mode.

| **Vascular territories** | **N** | **Basic mode** | | | **Enhanced mode** | | |
| --- | --- | --- | --- | --- | --- | --- | --- |
|  |  | **TP** | **FN** | **Sensitivity** | **TP** | **FN** | **Sensitivity** |
| 1 | 25 | 18 | 7 | 72.0% **(50.6–87.9)** | 21 | 4 | 84.0% **(63.9–95.5)** |
| 2 | 25 | 23 | 2 | 92.0% **(74.0–99.0)** | 24 | 1 | 96.0% **(79.6–99.9)** |
| 3 | 14 | 14 | 0 | 100.0% **(76.8–100.0)** | 14 | 0 | 100.0% **(76.8–100.0)** |
| 4 | 12 | 12 | 0 | 100.0% **(73.5–100.0)** | 12 | 0 | 100.0% **(73.5–100.0)** |
| Total | 76 | 67 | 9 | 88.2% **(78.7–94.4)** | 71 | 5 | 93.4% **(85.3–97.8)** |

**Table S3**. Model sensitivity on the prospective test set (n = 150) stratified by the number of vascular territories with confirmed atherosclerotic plaque. TP: true positive; FN: false negative.

**Table S4. Inconclusive result analysis**

AItheroscope incorporates a confidence assessment mechanism that flags predictions falling within a narrow band around the decision threshold as inconclusive, rather than returning a potentially unreliable binary classification. To evaluate the effect of this mechanism on model performance, analyses were repeated after excluding the 5% and 10% of predictions closest to the F1-optimized decision threshold. As shown in Supplementary Table 2, excluding uncertain predictions resulted in progressive improvement across all performance metrics in both modes, confirming that the confidence assessment mechanism effectively identifies cases where model predictions are least reliable.

| **Mode** | **% Inconclusive** | **N analyzed** | **AUC** | **Sensitivity** | **Specificity** | **PPV** | **NPV** |
| --- | --- | --- | --- | --- | --- | --- | --- |
| Basic | 0% | 150 | 0.80 **(0.73–0.87)** | 88.2% **(78.7–94.4)** | 56.8% **(44.7–68.2)** | 67.7% **(57.5–76.7)** | 82.4% **(69.1–91.6)** |
|  | 5% | 142 | 0.81 **(0.74–0.88)** | 90.3% **(81.0–96.0)** | 60.0% **(47.6–71.5)** | 69.1% **(59.5–79.0)** | 85.7% **(72.8–94.1)** |
|  | 10% | 135 | 0.81 **(0.74–0.88)** | 91.2% **(81.8–96.7)** | 62.5% **(50.0–74.2)** | 70.5% **(60.6–80.5)** | 87.0% **(74.8–95.3)** |
| Enhanced | 0% | 150 | 0.86 **(0.80–0.92)** | 93.4% **(85.3–97.8)** | 64.9% **(52.9–75.6)** | 73.2% **(63.2–81.7)** | 90.6% **(79.3–96.9)** |
|  | 5% | 142 | 0.87 **(0.81–0.93)** | 94.4% **(86.4–98.5)** | 68.6% **(56.4–79.1)** | 75.6% **(65.4–84.0)** | 92.3% **(81.5–97.9)** |
|  | 10% | 135 | 0.88 **(0.82–0.94)** | 95.6% **(87.6–99.1)** | 73.1% **(60.9–83.2)** | 78.3% **(67.9–86.6)** | 94.2% **(84.1–98.8)** |

**Table S4.** Model performance after exclusion of inconclusive predictions. Inconclusive cases were defined as those with predicted probabilities closest to the F1-optimized decision threshold. AUC: area under the ROC curve; PPV: positive predictive value; NPV: negative predictive value.

**Table S5. Composition of the Vision Transformer pretraining corpus.**

**Publicly available retinal fundus image datasets used for the in-house self-supervised pretraining of the ViT encoder, prior to fine-tuning on the study cohort. All datasets are open-access and were used under their respective CC BY licenses.**

| **Dataset** | **Origin** | **Camera / device** | **Resolution (px)** | **Images** |
| --- | --- | --- | --- | --- |
| IDRiD (13) | **India (Nanded, Maharashtra)** | **Kowa VX-10α (50° FOV)** | **4288 × 2848** | **516** |
| RFMiD (14) | **India (Maharashtra)** | **Topcon 3D OCT-2000; Kowa VX-10α; Topcon TRC-NW300** | **2144 × 1424; 4288 × 2848; 2048 × 1536** | **3,200** |
| RFMiD 2.0 (15) | **India (Jalna / Nanded, Maharashtra)** | **Carl Zeiss FF450; Topcon TRC-NW300** | **2048 × 1536** | **860** |
| **Total** |  |  |  | **4,576** |
